# Associations between deep venous thrombosis and thyroid diseases: a two-sample bidirectional Mendelian randomization study

**DOI:** 10.1186/s40001-024-01933-1

**Published:** 2024-06-14

**Authors:** Lifeng Zhang, Kaibei Li, Qifan Yang, Yao Lin, Caijuan Geng, Wei Huang, Wei Zeng

**Affiliations:** 1 Department of Vascular Surgery, Hospital of Chengdu University of Traditional Chinese Medicine, No. 39, Shierqiao Road, Jinniu District, Chengdu, 610072 Sichuan People’s Republic of China; 2 Disinfection Supply Center, Hospital of Chengdu University of Traditional Chinese Medicine, No. 39, Shierqiao Road, Jin Niu District, Chengdu, 610072 Sichuan People’s Republic of China

**Keywords:** Deep venous thrombosis, Thyroid diseases, Mendelian randomization analysis, Database

## Abstract

**Background:**

Some previous observational studies have linked deep venous thrombosis (DVT) to thyroid diseases; however, the findings were contradictory. This study aimed to investigate whether some common thyroid diseases can cause DVT using a two-sample Mendelian randomization (MR) approach.

**Methods:**

This two-sample MR study used single nucleotide polymorphisms (SNPs) identified by the FinnGen genome-wide association studies (GWAS) to be highly associated with some common thyroid diseases, including autoimmune hyperthyroidism (962 cases and 172,976 controls), subacute thyroiditis (418 cases and 187,684 controls), hypothyroidism (26,342 cases and 59,827 controls), and malignant neoplasm of the thyroid gland (989 cases and 217,803 controls. These SNPs were used as instruments. Outcome datasets for the GWAS on DVT (6,767 cases and 330,392 controls) were selected from the UK Biobank data, which was obtained from the Integrative Epidemiology Unit (IEU) open GWAS project. The inverse variance weighted (IVW), MR-Egger and weighted median methods were used to estimate the causal association between DVT and thyroid diseases. The Cochran’s Q test was used to quantify the heterogeneity of the instrumental variables (IVs). MR Pleiotropy RESidual Sum and Outlier test (MR-PRESSO) was used to detect horizontal pleiotropy. When the causal relationship was significant, bidirectional MR analysis was performed to determine any reverse causal relationships between exposures and outcomes.

**Results:**

This MR study illustrated that autoimmune hyperthyroidism slightly increased the risk of DVT according to the IVW [odds ratio (OR) = 1.0009; *p* = 0.024] and weighted median methods [OR = 1.001; *p* = 0.028]. According to Cochran’s Q test, there was no evidence of heterogeneity in IVs. Additionally, MR-PRESSO did not detect horizontal pleiotropy (*p* = 0.972). However, no association was observed between other thyroid diseases and DVT using the IVW, weighted median, and MR-Egger regression methods.

**Conclusions:**

This study revealed that autoimmune hyperthyroidism may cause DVT; however, more evidence and larger sample sizes are required to draw more precise conclusions.

**Supplementary Information:**

The online version contains supplementary material available at 10.1186/s40001-024-01933-1.

## Introduction

Deep venous thrombosis (DVT) is a common type of disease that occurs in 1–2 individuals per 1000 each year [1]. In the post-COVID-19 era, DVT showed a higher incidence rate [2]. Among hospitalized patients, the incidence rate of this disease was as high as 2.7% [3], increasing the risk of adverse events during hospitalization. According to the Registro Informatizado Enfermedad Tromboembolica (RIETE) registry, which included data from ~ 100,000 patients from 26 countries, the 30-day mortality rate was 2.6% for distal DVT and 3.3% for proximal DVT [4]. Other studies have shown that the one-year mortality rate of DVT is 19.6% [5]. DVT and pulmonary embolism (PE), collectively referred to as venous thromboembolism (VTE), constitute a major global burden of disease [6].

Thyroid diseases are common in the real world. Previous studies have focused on the relationship between DVT and thyroid diseases, including thyroid dysfunction and thyroid cancer. Some case reports [7–9] have demonstrated that hyperthyroidism is often associated with DVT and indicates a worse prognosis [10]. The relationship between thyroid tumors and venous thrombosis has troubled researchers for many years. In 1989, the first case of papillary thyroid carcinoma presenting with axillary vein thrombosis as the initial symptom was reported [11]. In 1995, researchers began to notice the relationship between thyroid tumors and hypercoagulability [12], laying the foundation for subsequent extensive research. However, the aforementioned observational studies had limitations, such as small sample sizes, selection bias, reverse causality, and confounding factors, which may have led to unreliable conclusions [13].

Previous studies have explored the relationship of thyroid disease and DVT and revealed that high levels of thyroid hormones may increase the risk of DVT. Hyperthyroidism promotes a procoagulant and hypofibrinolytic state by affecting the von Willebrand factor, factors VIII, IV, and X, fibrinogen, and plasminogen activator inhibitor-1 [14, 15]. At the molecular level, researchers believe that thyroid hormones affect coagulation levels through an important nuclear thyroid hormone receptor (TR), TRβ [16], and participate in pathological coagulation through endothelial dysfunction. Thyroid hormones may have non-genetic effects on the behavior of endothelial cells [17, 18]. In a study regarding tumor thrombosis, Lou [19] found that 303 circular RNAs were differentially expressed in DVT using microarray. Kyoto Encyclopedia of Genes and Genomes (KEGG) analysis revealed that the most significantly enriched pathways included thyroid hormone-signaling pathway and endocytosis, and also increased level of proteoglycans in cancer. This indicated that tumor cells and thyroid hormones might interact to promote thrombosis. Based on these studies, we speculated that thyroid diseases, including thyroid dysfunction and thyroid tumors, may cause DVT.

Mendelian randomization (MR) research is a causal inference technique that can be used to assess the causal relationship and reverse causation between specific exposure and outcome factors. If certain assumptions [20] are fulfilled, genetic variants can be employed as instrumental variables (IVs) to establish causal relationships. Bidirectional MR analysis can clarify the presence of reverse causal relationships [21], making the conclusions more comprehensive. Accordingly, we aimed to apply a two-sample MR strategy to investigate whether DVT is related to four thyroid diseases, including autoimmune hyperthyroidism, subacute thyroiditis, hypothyroidism, and thyroid cancer.

## Methods

### Study design

**Fig. 1:**
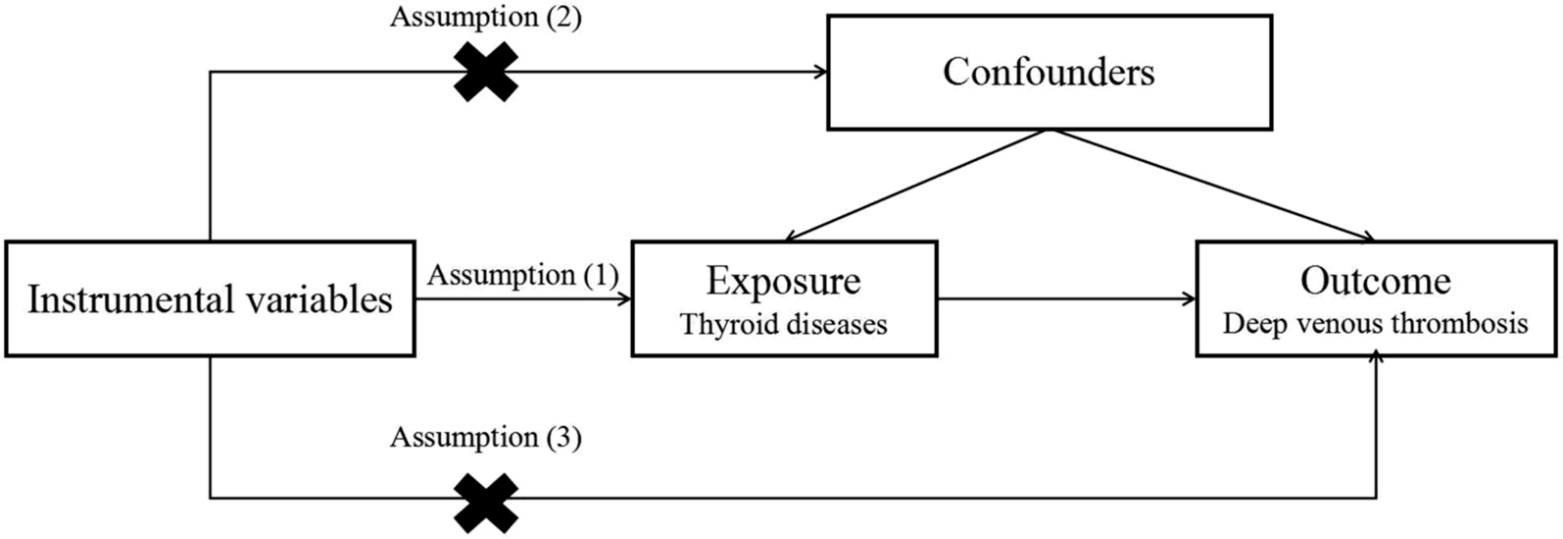
The relationship between instrumental variables, exposure, outcome, and confounding factors

MR relies on single nucleotide polymorphisms (SNPs) as IVs. The IVs should fulfill the following three criteria [22]: (1) IVs should be strongly associated with exposure. (2) Genetic variants must be independent of unmeasured confounding factors that may affect the exposure–outcome association. (3) IVs are presumed to affect the outcome only through their associations with exposure (Fig. 1). IVs that met the above requirements were used to estimate the relationship between exposure and outcome. Our study protocol conformed to the STROBE-MR Statement [23], and all methods were performed in accordance with the relevant guidelines and regulations.

### Data sources and instruments

Datasets (Table 1) in this study were obtained from a publicly available database (the IEU open genome-wide association studies (GWAS) project [24] (https://gwas.mrcieu.ac.uk)). There was no overlap in samples between the data sources of outcome and exposures. Using de-identified summary-level data, privacy information such as overall age and gender were hidden. Ethical approval was obtained for all original work. This study complied with the terms of use of the database.

**Table 1:** Genetic summary data sources of exposures and outcome

Dataset	Trait	Sample size	Ncase	Ncontrol	Population	GWAS ID	Dataset link
Exposures	Autoimmune hyperthyroidism	173,938	962	172,976	European	finn-b-AUTOIMMUNE_HYPERTHYROIDISM	https://gwas.mrcieu.ac.uk/datasets/finn-b-AUTOIMMUNE_HYPERTHYROIDISM/
Exposures	Subacute thyroiditis	188,102	418	187,684	European	finn-b-E4_THYROIDITSUBAC	https://gwas.mrcieu.ac.uk/datasets/finn-b-E4_THYROIDITSUBAC/
Exposures	Hypothyroidism	86,169	26,342	59,827	European	finn-b-HYPOTHYROIDISM	https://gwas.mrcieu.ac.uk/datasets/finn-b-HYPOTHYROIDISM/
Exposures	Malignant neoplasm of thyroid gland	218,792	989	217,803	European	finn-b-C3_THYROID_GLAND	https://gwas.mrcieu.ac.uk/datasets/finn-b-C3_THYROID_GLAND/
Outcome	Deep venous thrombosis	337,159	6,767	330,392	European	ukb-a-65	https://gwas.mrcieu.ac.uk/datasets/ukb-a-65/

MR analysis was performed using the R package “TwoSampleMR”. SNPs associated with each thyroid disease at the genome-wide significance threshold of *p* < 5.0 × 10^–8^ were selected as potential IVs. To ensure independence between the genetic variants used as IVs, the linkage disequilibrium (LD) threshold for grouping was set to r^2^ < 0.001 with a window size of 10,000 kb. The SNP with the lowest *p*-value at each locus was retained for analyses.

### Statistical analysis

Multiple MR methods were used to infer causal relationships between thyroid diseases and DVT, including the inverse variance weighted (IVW), weighted median, and MR-Egger tests, after harmonizing the SNPs across the GWASs of exposures and outcomes. The main analysis was conducted using the IVW method. Heterogeneity and pleiotropy were also performed in each MR analysis. Meanwhile, the MR-PRESSO Global test [25] was utilized to detect horizontal pleiotropy. The effect trend of SNP was observed through a scatter plot, and the forest plot was used to observe the overall effects. When a significant causal relationship was confirmed by two-sample MR analysis, bidirectional MR analysis was performed to assess reverse causal relationships by swapping exposure and outcome factors. Parameters were set the same as before. All abovementioned statistical analyses were performed using the package TwoSampleMR (version 0.5.7) in the R program (version 4.2.1).

## Results

**Figure 2:**
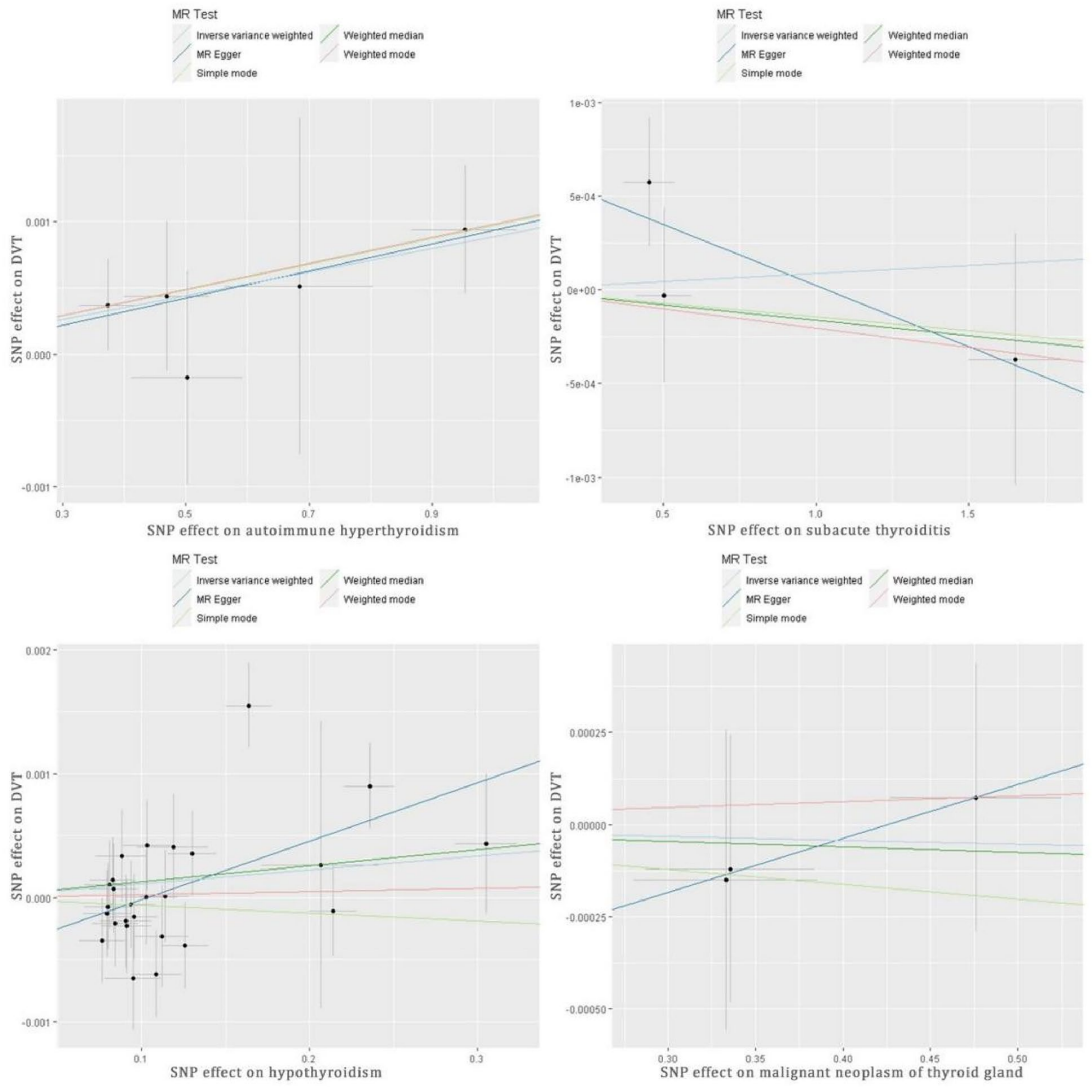
The estimated scatter plot of the association between thyroid diseases and DVT. MR-analyses are derived using IVW, MR-Egger, weighted median and mode. By fitting different models, the scatter plot showed the relationship between SNP and exposure factors, predicting the association between SNP and outcomes

After harmonizing the SNPs across the GWASs for exposures and outcomes, the IVW (OR = 1.0009, *p* = 0.024, Table 2) and weighted median analyses (OR = 1.001, *p* = 0.028) revealed significant causal effects between autoimmune hyperthyroidism and DVT risk. Similar results were observed using the weighted median approach Cochran’s Q test, MR-Egger intercept, and MR-PRESSO tests suggested that the results were not influenced by pleiotropy and heterogeneity (Table 2). However, the leave-one-out analysis revealed a significant difference after removing some SNPs (rs179247, rs6679677, rs72891915, and rs942495, *p* < 0.05, Figure S2a), indicating that MR results were dependent on these SNPs (Figure S2, Table S1). No significant effects were observed in other thyroid diseases (Table 2). The estimated scatter plot of the association between thyroid diseases and DVT is presented in Fig. 2, indicating a positive causal relationship between autoimmune hyperthyroidism and DVT (Fig. 2a). The forest plots of single SNPs affecting the risk of DVT are displayed in Figure S1.

**Table 2:** MR results related to DVT and thyroid diseases

Methods	No. of SNPs	MR analysis results		Heterogeneity		Pleiotropy			
		OR	*p*	Cochran’s Q	*p*	MR-Egger intercept	*p*	MR-PRESSO Global Test	*p*
Autoimmune hyperthyroidism on DVT									
IVW	5	1.0009	0.024	0.649	0.958	–	–	0.919	0.972
MR-Egger	5	1.001	0.385	0.629	0.890	− 0.00008	0.896		
Weighted median	5	1.001	0.028	–	–	–	–		
Subacute thyroiditis on DVT									
IVW	3	1.00009	0.833	3.047	0.218	–	–	Not enough instrumental variables	Not enough instrumental variables
MR-Egger	3	0.999	0.480	0.983	0.321	0.0007	0.387		
Weighted median	3	0.999	0.674	–	–	–	–		
Hypothyroidism on DVT									
IVW	26	1.001	0.123	38.433	0.042	–	–	44.027	0.020
MR-Egger	26	1.005	0.013	31.943	0.128	− 0.0005	0.037		
Weighted median	26	1.001	0.162	–	–	–	–		
Malignant neoplasm of thyroid gland on DVT									
IVW	3	0.999	0.849	0.249	0.883	–	–	Not enough instrumental variables	Not enough instrumental variables
MR-Egger	3	1.001	0.728	0.002	0.964	− 0.0006	0.707		
Weighted median	3	0.999	0.802	–	–	–	–

Bidirectional MR analysis was performed to further determine the relationship between autoimmune hyperthyroidism and DVT. The reverse causal relationship was not observed (Table S2), which indicated that autoimmune hyperthyroidism can cause DVT from a mechanism perspective.

## Discussion

This study used MR to assess whether thyroid diseases affect the incidence of DVT. The results showed that autoimmune hyperthyroidism can increase the risk of DVT occurrence, but a reverse causal relationship was not observed between them using bidirectional MR analysis. However, other thyroid diseases, such as subacute thyroiditis, hypothyroidism, and thyroid cancer, did not show a similar effect.

Recently, several studies have suggested that thyroid-related diseases may be associated with the occurrence of DVT in the lower extremities, which provided etiological clues leading to the occurrence of DVT in our subsequent research. In 2006, a review mentioned the association between thyroid dysfunction and coagulation disorders [26], indicating a hypercoagulable state in patients with hyperthyroidism. In 2011, a review further suggested a clear association between hypothyroidism and bleeding tendency, while hyperthyroidism appeared to increase the risk of thrombotic events, particularly cerebral venous thrombosis [27]. A retrospective cohort study [28] supported this conclusion, but this study only observed a higher proportion of concurrent thyroid dysfunction in patients with cerebral venous thrombosis. The relationship between thyroid function and venous thromboembolism remains controversial. Krieg VJ et al. [29] found that hypothyroidism has a higher incidence rate in patients with chronic thromboembolic pulmonary hypertension and may be associated with more severe disease, which seemed to be different from previous views that hyperthyroidism may be associated with venous thrombosis. Alsaidan [30] also revealed that the risk of developing venous thrombosis was almost increased onefold for cases with a mild-to-moderate elevation of thyroid stimulating hormone and Free thyroxine 4(FT4). In contrast, it increased twofold for cases with a severe elevation of thyroid stimulating hormone and FT4. Raised thyroid hormones may increase the synthesis or secretion of coagulation factors or may decrease fibrinolysis, which may lead to the occurrence of coagulation abnormality.

Other thyroid diseases are also reported to be associated with DVT. In a large prospective cohort study [31], the incidence of venous thromboembolism was observed to increase in patients with thyroid cancer over the age of 60. However, other retrospective studies did not find any difference compared with the general population [32]. In the post-COVID-19 era, subacute thyroiditis has received considerable attention from researchers. New evidence suggests that COVID-19 may be associated with subacute thyroiditis [33, 34]. Mondal et al. [35] found that out of 670 COVID-19 patients, 11 presented with post-COVID-19 subacute thyroiditis. Among them, painless subacute thyroiditis appeared earlier and exhibited symptoms of hyperthyroidism. Another case report also indicated the same result, that is, subacute thyroiditis occurred after COVID-19 infection, accompanied by thyroid function changes [36]. This led us to hypothesize that subacute thyroiditis may cause DVT through alterations in thyroid function.

This study confirmed a significant causal relationship between autoimmune hyperthyroidism and DVT (*p* = 0.02). The data were tested for heterogeneity and gene pleiotropy using MR-Egger, Cochran’s Q, and MR-PRESSO tests. There was no evidence that the results were influenced by pleiotropy or heterogeneity. In the leave-one-out analysis, four of the five selected SNPs showed significant effects of autoimmune hyperthyroidism on DVT, suggesting an impact of these SNPs on DVT outcome. Previous studies have focused on the relationship between hyperthyroidism and its secondary arrhythmias and arterial thromboembolism [37, 38]. This study emphasized the risk of DVT in patients with hyperthyroidism, which has certain clinical implications. Prophylactic anticoagulant therapy was observed to help prevent DVT in patients with hyperthyroidism. Unfortunately, the results of this study did not reveal any evidence that suggests a relationship between other thyroid diseases and DVT occurrence. This may be due to the limited database, as this study only included the GWAS data from a subset of European populations. Large-scale multiracial studies are needed in the future.

There are some limitations to this study. First, it was limited to participants of European descent. Consequently, further investigation is required to confirm these findings in other ethnicities. Second, this study did not reveal the relationship between complications of hyperthyroidism and DVT. Additionally, this study selected IVs from the database using statistical methods rather than selecting them from the real population. This may result in weaker effects of the screened IVs and reduce the clinical significance of MR analysis. Moreover, the definitions of some diseases in this study were not clear in the original database, and some of the diseases were self-reported, which may reduce the accuracy of diagnosis. Further research is still needed to clarify the causal relationship between DVT and thyroid diseases based on prospective cohort and randomized controlled trials (RCTs).

## Conclusions

This study analyzed large-scale genetic data and provided evidence of a causal relationship between autoimmune hyperthyroidism and the risk of DVT, Compared with the other thyroid diseases investigated. Prospective RCTs or MR studies with larger sample sizes are still needed to draw more precise conclusions.

### Supplementary Information

Additional file 1.

## Data Availability

The IEU open gwas project, https://gwas.mrcieu.ac.uk/
