# Supplementary material for: Associations between deep venous thrombosis and thyroid diseases: a two-sample bidirectional Mendelian randomization study
Source: Eur J Med Res. 2024 Jun 14;29:327. doi: 10.1186/s40001-024-01933-1 (PMC11177513; doi:10.1186/s40001-024-01933-1)
Supplement: Supplementary file 1 — Additional file 1. [file 40001_2024_1933_MOESM1_ESM.docx]

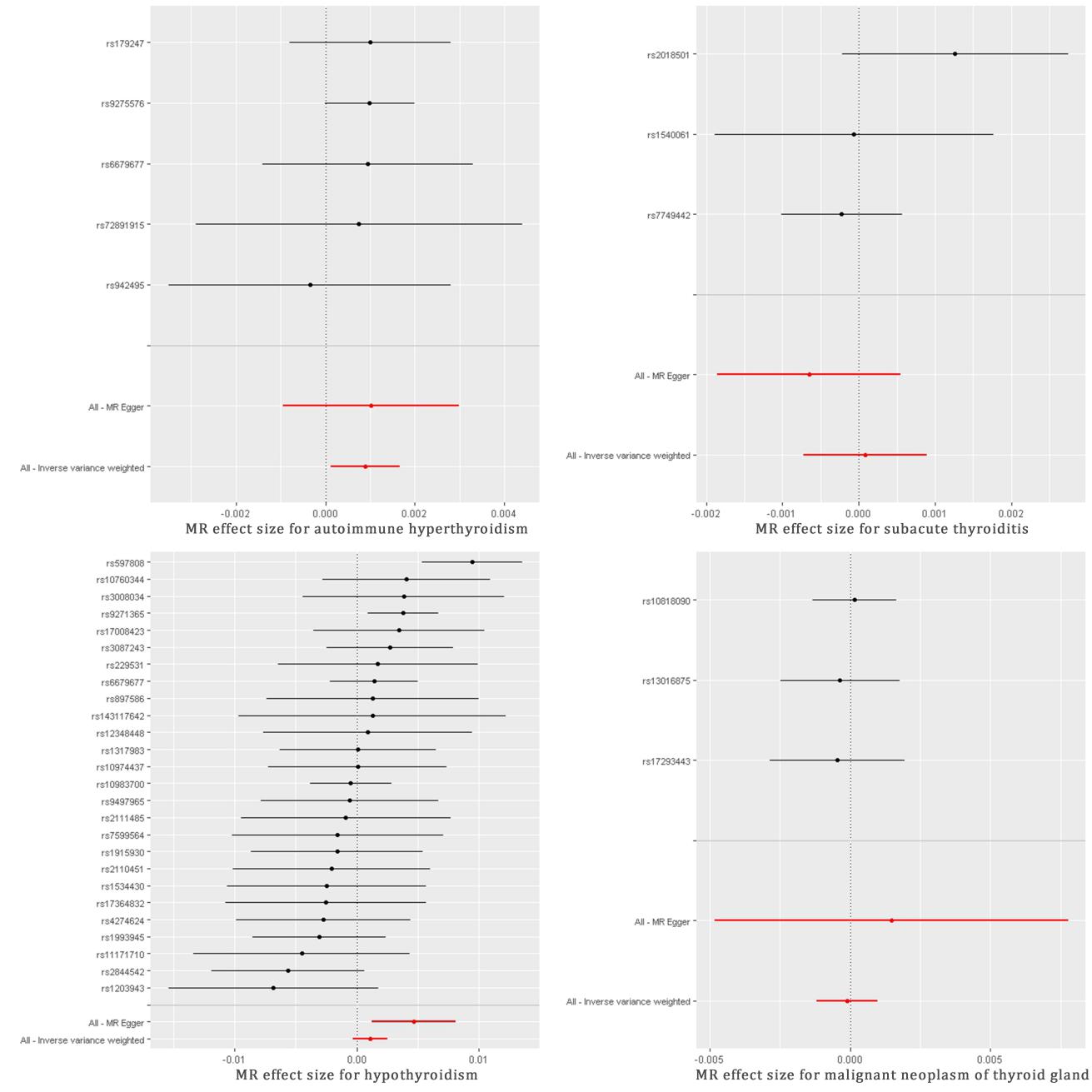


Figure S1. The forest plots of single SNPs affecting the risk of DVT

The forest plot displayed the effect size and 95% confidence interval for each SNP.


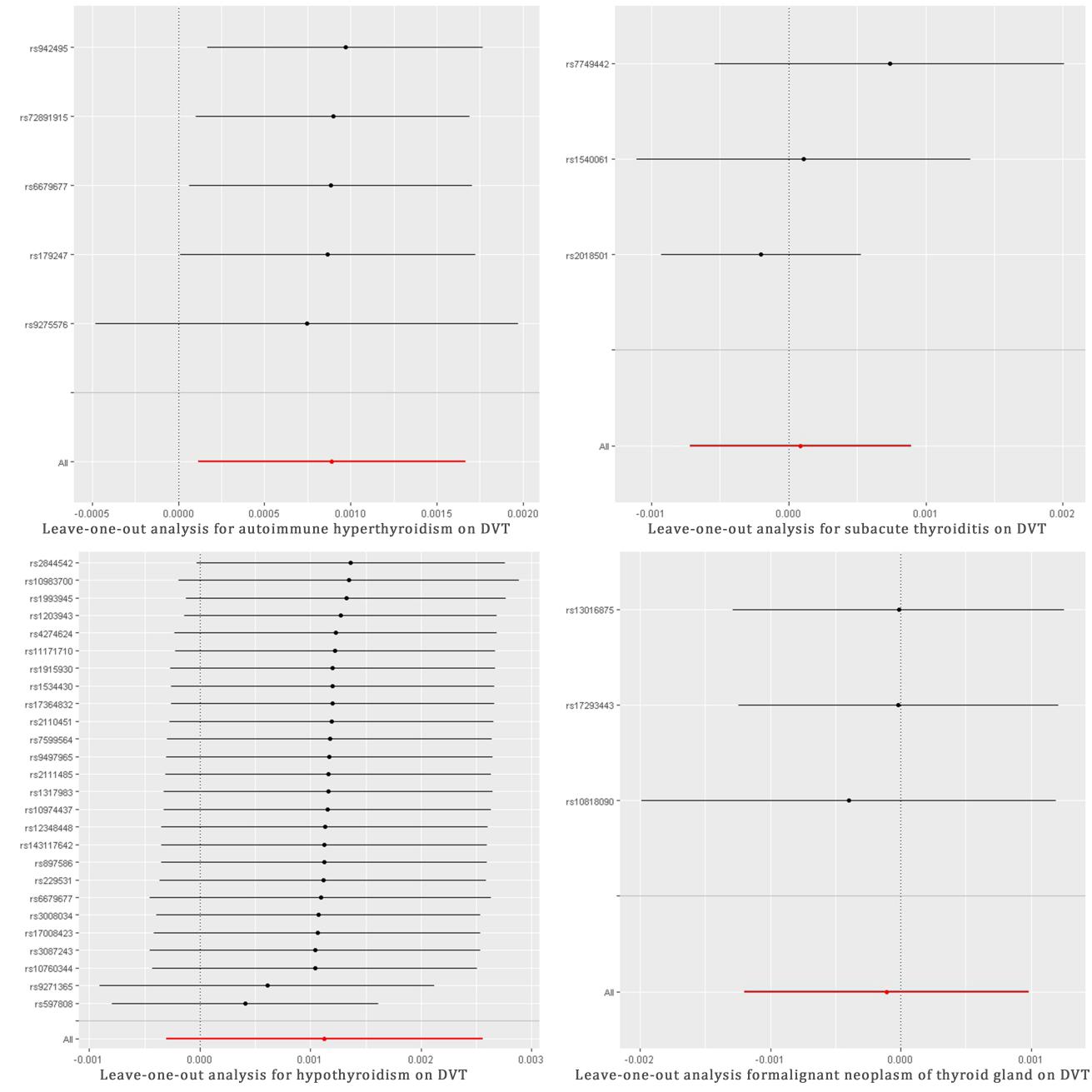


Figure S2. Leave-one-out sensitivity analysis for thyroid diseases on DVT

The forest plot generated by the leave-one-out method showed the estimated results of MR when removing each individual SNP. If the result changed significantly after removing a SNP, it indicated that the SNP had a large impact on the result, reducing the reliability of the analysis results.

| Autoimmune hyperthyroidism on DVT | | | |
| --- | --- | --- | --- |
| SNP | b | se | p |
| rs179247 | 0.000867183 | 0.000437712 | 0.04757213 |
| rs6679677 | 0.000884952 | 0.000418735 | 0.03456703 |
| rs72891915 | 0.00089717 | 0.000404592 | 0.0265911 |
| rs9275576 | 0.000746588 | 0.000626572 | 0.23344025 |
| rs942495 | 0.000969907 | 0.000407785 | 0.01738441 |
| All | 0.000890436 | 0.000395343 | 0.02430269 |
| Subacute thyroiditis on DVT | | | |
| SNP | b | se | p |
| rs1540061 | 0.000108883 | 0.000620315 | 0.8606648 |
| rs2018501 | -0.000199085 | 0.000371829 | 0.5923591 |
| rs7749442 | 0.000737815 | 0.000649634 | 0.2560652 |
| All | 8.66314E-05 | 0.000411678 | 0.8333283 |
| Hypothyroidism on DVT | | | |
| SNP | b | se | p |
| rs10760344 | 0.001040578 | 0.000749614 | 0.16509116 |
| rs10974437 | 0.001153369 | 0.0007547 | 0.12645104 |
| rs10983700 | 0.001347148 | 0.000784674 | 0.08601115 |
| rs11171710 | 0.001223493 | 0.000736902 | 0.09685039 |
| rs1203943 | 0.00127289 | 0.000719299 | 0.07678971 |
| rs12348448 | 0.001130736 | 0.000752905 | 0.13314069 |
| rs1317983 | 0.001161723 | 0.000757566 | 0.12515397 |
| rs143117642 | 0.001124928 | 0.000750202 | 0.13374389 |
| rs1534430 | 0.001199823 | 0.000746186 | 0.10784808 |
| rs17008423 | 0.001061885 | 0.000752071 | 0.15796503 |
| rs17364832 | 0.001199711 | 0.000745999 | 0.10779256 |
| rs1915930 | 0.001202127 | 0.000750413 | 0.10916592 |
| rs1993945 | 0.001322031 | 0.000739418 | 0.07378641 |
| rs2110451 | 0.001193364 | 0.000747702 | 0.11047954 |
| rs2111485 | 0.00116378 | 0.000750807 | 0.12113216 |
| rs229531 | 0.001114191 | 0.000753354 | 0.13914732 |
| rs2844542 | 0.001365139 | 0.000711689 | 0.0550897 |
| rs3008034 | 0.001072378 | 0.000749243 | 0.15234944 |
| rs3087243 | 0.00104192 | 0.0007615 | 0.17123478 |
| rs4274624 | 0.001230724 | 0.000744442 | 0.0982868 |
| rs597808 | 0.000408923 | 0.000614412 | 0.50569766 |
| rs6679677 | 0.001091251 | 0.000787096 | 0.1656169 |
| rs7599564 | 0.001175163 | 0.000749057 | 0.11668144 |
| rs897586 | 0.00112343 | 0.000752728 | 0.13557375 |
| rs9271365 | 0.000608815 | 0.000771182 | 0.42984441 |
| rs9497965 | 0.001170376 | 0.000753467 | 0.12034618 |
| All | 0.001126647 | 0.000730977 | 0.12324629 |
| Malignant neoplasm of thyroid gland on DVT | | | |
| SNP | b | se | p |
| rs10818090 | -0.000398358 | 0.00081146 | 0.6234868 |
| rs13016875 | -1.4744E-05 | 0.000649276 | 0.9818829 |
| rs17293443 | -1.62949E-05 | 0.00062583 | 0.9792276 |
| All | -0.000105765 | 0.000557104 | 0.8494277 |

Table S1. Single SNP effect in Leave-one-out sensitivity analysis

| Methods | No. of SNPs | MR analysis results | | Heterogeneity | | Pleiotropy | | | |
| --- | --- | --- | --- | --- | --- | --- | --- | --- | --- |
|  |  | OR | p | Cochran’s Q | p | MR-Egger intercept | p | MR-PRESSO Global Test | p |
| DVT on Autoimmune hyperthyroidism | | | | | | | | | |
| IVW | 8 | 0.0009 | 0.117 | 7.883 | 0.343 | - | - | 9.081 | 0.501 |
| MR-Egger | 8 | 0.0007 | 0.321 | 7.881 | 0.247 | 0.002 | 0.966 |  |  |
| Weighted median | 8 | 0.0011 | 0.157 | - | - | - | - |  |  |

Table S2. Bidirectional Mendelian randomization analysis of DVT and Autoimmune Hyperthyroidis

| **Item No.** | **Section** | **Checklist item** | **Relevant text from manuscript** |
| --- | --- | --- | --- |
| 1 | **TITLE and ABSTRACT** | Indicate Mendelian randomization (MR) as the study’s design in the title and/or the abstract if that is a main purpose of the study | The title of this manuscript has described the study as an MR study. |
|  | **INTRODUCTION** |  |  |
| 2 | **Background** | Explain the scientific background and rationale for the reported study. What is the exposure? Is a potential causal relationship between exposure and outcome plausible? Justify why MR is a helpful method to address the study question | Exposures: Thyroid diseases; Outcome: DVT  Previous studies have reported an association between thyroid diseases and DVT, but most of these studies were observational in nature and were unable to establish a causal relationship. Consequently, it is necessary to use the MR approach to investigate whether the exposure factors mentioned above are associated with outcomes, ultimately achieving the goal of preventing or delaying disease progression. |
| 3 | **Objectives** | State specific objectives clearly, including pre-specified causal hypotheses (if any). State that MR is a method that, under specific assumptions, intends to estimate causal effects | Using dual-sample MR to explore the association between thyroid diseases and DVT. |
|  | **METHODS** |  |  |
| 4 | **Study design and data sources** | Present key elements of the study design early in the article. Consider including a table listing sources of data for all phases of the study. For each data source contributing to the analysis, describe the following: |  |
|  | a) | Setting: Describe the study design and the underlying population, if possible. Describe the setting, locations, and relevant dates, including periods of recruitment, exposure, follow-up, and data collection, when available. | We used Genome-Wide Association Studies (GWAS) from UK Biobank data and the FinnGen GWAS, as shown in Table 1. |
|  | b) | Participants: Give the eligibility criteria, and the sources and methods of selection of participants. Report the sample size, and whether any power or sample size calculations were carried out prior to the main analysis | The datasets (Table 1) in this study were obtained from the public database, the IEU open GWAS project [11] (https://gwas.mrcieu.ac.uk/). The sample size is presented in Table 1. Methods for calculating the sample size are not reported. |
|  | c) | Describe measurement, quality control and selection of genetic variants | We used deidentified summary-level data, and this study complies with the terms of use of the database. The original research considered the measurement, quality control, and selection of genetic variants. |
|  | d) | For each exposure, outcome, and other relevant variables, describe methods of assessment and diagnostic criteria for diseases | Considered in the original research. |
|  | e) | Provide details of ethics committee approval and participant informed consent, if relevant | Considered in the original research. |
| 5 | **Assumptions** | Explicitly state the three core IV assumptions for the main analysis (relevance, independence and exclusion restriction) as well assumptions for any additional or sensitivity analysis | Three core assumptions should be satisfied: (1) instrumental variables (IVs) should be strongly associated with exposure. (2) Genetic variants must be independent of unmeasured confounding factors that may affect the exposure-outcome association. (3) IVs are presumed to affect the outcome only through their associations with exposure (Figure 1). |
| 6 | **Statistical methods: main analysis** | Describe statistical methods and statistics used |  |
|  | a) | Describe how quantitative variables were handled in the analyses (i.e., scale, units, model) | There was no conversion of the statistical effect size or measurement unit related to exposure and outcome; therefore, it was not reported. |
|  | b) | Describe how genetic variants were handled in the analyses and, if applicable, how their weights were selected | We used several MR approaches to estimate the causal effects, including the inverse variance weighted (IVW), weighted median, and MR-Egger approaches, after harmonizing the SNPs across GWASs of exposures and outcomes. The primary analysis was conducted using the IVW method. |
|  | c) | Describe the MR estimator (e.g. two-stage least squares, Wald ratio) and related statistics. Detail the included covariates and, in case of two-sample MR, whether the same covariate set was used for adjustment in the two samples | We used GWAS from the UK Biobank data and the FinnGen GWAS. The original study did not use genetic models to adjust for factors such as age, gender, and study location. |
|  | d) | Explain how missing data were addressed | Considered in the original research. |
|  | e) | If applicable, indicate how multiple testing was addressed | This study used a two-sample MR approach and did not involve multiple testing. |
| 7 | **Assessment of assumptions** | Describe any methods or prior knowledge used to assess the assumptions or justify their validity | Previous studies have observed a potential association between the exposure and outcome, similar to this study. There are no sample duplicates in the exposure and outcome dataset. Furthermore, due to the large number of GWAS data included in this study, large-scale summary statistics have become more widely available, enabling two-sample MR analysis with significantly improved statistical power. |
| 8 | **Sensitivity analyses and additional analyses** | Describe any sensitivity analyses or additional analyses performed (e.g. comparison of effect estimates from different approaches, independent replication, bias analytic techniques, validation of instruments, simulations) | Heterogeneity and pleiotropy were also performed using Cochran’s Q test, MR-Egger intercept and MR-PRESSO Global Test. |
| 9 | **Software and pre-registration** |  |  |
|  | a) | Name statistical software and package(s), including version and settings used | All statistical analyses above were performed using the package TwoSampleMR (version 0.5.7) in the R program (version 4.2.1). |
|  | b) | State whether the study protocol and details were pre-registered (as well as when and where) | The study protocol and details were not pre-registered. |
|  | **RESULTS** |  |  |
| 10 | **Descriptive data** |  |  |
|  | a) | Report the numbers of individuals at each stage of included studies and reasons for exclusion. Consider use of a flow diagram | The original study did not specify the reasons for excluding subjects. The numbers of individuals at each stage of included studies are shown in Table 1 |
|  | b) | Report summary statistics for phenotypic exposure(s), outcome(s), and other relevant variables (e.g. means, SDs, proportions) | The original study did not specify the summary statistics. |
|  | c) | If the data sources include meta-analyses of previous studies, provide the assessments of heterogeneity across these studies | N/A |
|  | d) | For two-sample MR:  i.  Provide justification of the similarity of the genetic variant-exposure associations between the exposure and outcome samples  ii.  Provide information on the number of individuals who overlap between the exposure and outcome studies | The samples used in this study were all from Europe; accordingly, there is very little racial heterogeneity, and the original study did not report overlapping population information. |
| 11 | **Main results** |  |  |
|  | a) | Report the associations between genetic variant and exposure, and between genetic variant and outcome, preferably on an interpretable scale | Presented in Table S1 |
|  | b) | Report MR estimates of the relationship between exposure and outcome, and the measures of uncertainty from the MR analysis, on an interpretable scale, such as odds ratio or relative risk per SD difference | Indicated in Table 2 |
|  | c) | If relevant, consider translating estimates of relative risk into absolute risk for a meaningful time period | N/A |
|  | d) | Consider plots to visualize results (e.g. forest plot, scatterplot of associations between genetic variants and outcome versus between genetic variants and exposure) | Displayed in Figures 2 and S1 |
| 12 | **Assessment of assumptions** |  |  |
|  | a) | Report the assessment of the validity of the assumptions | Presented in Table 2. We used Cochran’s Q test to assess the validity of the assumptions. |
|  | b) | Report any additional statistics (e.g., assessments of heterogeneity across genetic variants, such as *I^2^*, Q statistic or E-value) | Additional statistics are shown in Table 2. |
| 13 | **Sensitivity analyses and additional analyses** |  |  |
|  | a) | Report any sensitivity analyses to assess the robustness of the main results to violations of the assumptions | Heterogeneity and pleiotropy were also performed using Cochran’s Q test, MR-Egger intercept, and MR-PRESSO Global Test. The results are shown in Table 2. |
|  | b) | Report results from other sensitivity analyses or additional analyses |  |
|  | c) | Report any assessment of direction of causal relationship (e.g., bidirectional MR) | We used bidirectional MR to assess the direction of the causal relationship(Table S2) |
|  | d) | When relevant, report and compare with estimates from non-MR analyses | N/A |
|  | e) | Consider additional plots to visualize results (e.g., leave-one-out analyses) | Additional result plots are depicted in Figure S2 |
|  | **DISCUSSION** |  |  |
| 14 | **Key results** | Summarize key results with reference to study objectives | The results supported that autoimmune hyperthyroidism can increase the risk of DVT occurrence, and we did not find a reverse causal relationship between autoimmune hyperthyroidism and DVT using bidirectional MR analysis. |
| 15 | **Limitations** | Discuss limitations of the study, taking into account the validity of the IV assumptions, other sources of potential bias, and imprecision. Discuss both direction and magnitude of any potential bias and any efforts to address them | There are some limitations to this study. First, our study is limited to participants of European descent. Therefore, further investigation is needed to confirm these findings in other ethnicities. Second, this study did not clarify the relationship between complications of hyperthyroidism and DVT. Further research is required to supplement these details. Additionally, this study selected IVs from the database using statistical methods rather than selecting them in the real population, which may result in weaker effects of the screened IVs and reduce the clinical significance of MR analysis. Further studies should construct relevant cohorts and detect GWAS for targeted prospective research. |
| 16 | **Interpretation** |  |  |
|  | a) | Meaning: Give a cautious overall interpretation of results in the context of their limitations and in comparison with other studies | We discussed the relevant content and compared multiple previous studies to provide a reasonable interpretation of the results of this study. |
|  | b) | Mechanism: Discuss underlying biological mechanisms that could drive a potential causal relationship between the investigated exposure and the outcome, and whether the gene-environment equivalence assumption is reasonable. Use causal language carefully, clarifying that IV estimates may provide causal effects only under certain assumptions | Raised thyroid hormones may increase the synthesis or secretion of coagulation factors, or may decrease fibrinolysis, which may lead to the occurrence of coagulation abnormality. |
|  | c) | Clinical relevance: Discuss whether the results have clinical or public policy relevance, and to what extent they inform effect sizes of possible interventions | Our research confirmed a significant causal relationship between autoimmune hyperthyroidism and DVT. It is recommended to pay special attention to the assessment of DVT in patients with hyperthyroidism and provide timely treatment. |
| 17 | **Generalizability** | Discuss the generalizability of the study results (a) to other populations, (b) across other exposure periods/timings, and (c) across other levels of exposure | This study is limited to participants of European descent. Although it reduces potential biases caused by population structure, it limits the generalizability of MR results to other populations. |
|  | **OTHER INFORMATION** |  |  |
| 18 | **Funding** | Describe sources of funding and the role of funders in the present study and, if applicable, sources of funding for the databases and original study or studies on which the present study is based | N/A |
| 19 | **Data and data sharing** | Provide the data used to perform all analyses or report where and how the data can be accessed, and reference these sources in the article. Provide the statistical code needed to reproduce the results in the article, or report whether the code is publicly accessible and if so, where | Datasets (Table 1) in this study waswere obtained on the publicfrom a publicly available database (the IEU open gwasgenome-wide association studies (GWAS) project) |
| 20 | **Conflicts of Interest** | All authors should declare all potential conflicts of interest | N/A |

Table S3. STROBE-MR checklist of recommended items to address in reports of Mendelian randomization studies
